# Cephalometric evaluation of the airway space and hyoid bone in children with normal and atypical deglutition: correlation study

**DOI:** 10.1590/S1516-31802012000400006

**Published:** 2012-09-04

**Authors:** Almiro José Machado, Agrício Nubiato Crespo

**Affiliations:** I DDS. Researcher, Discipline of Otorhinolaryngology, Faculdade de Ciências Médicas da Universidade Estadual de Campinas (FCM-Unicamp), Campinas, São Paulo, Brazil.; II PhD. Otorhinolaryngologist, Discipline of Otorhinolaryngology, Faculdade de Ciências Médicas da Universidade Estadual de Campinas (FCM-Unicamp), Campinas, São Paulo, Brazil.

**Keywords:** Cephalometry, Deglutition, Hyoid bone, Oropharynx, Mouth, Circunferência craniana, Deglutição, Osso hióide, Orofaringe, Boca

## Abstract

**CONTEXT AND OBJECTIVE:**

Although there is a close relationship between swallowing and breathing, there are no studies evaluating the radiographic anatomy of the airway and its possible correlation with the radiographic position of the hyoid bone. The aim of this study was to evaluate the possible correlation of the radiographic position of the hyoid bone and airway space (PAS) in lateral radiographs on children with atypical deglutition, in comparison with those with normal swallowing.

**DESIGN AND SETTING:**

Cross-sectional analytical study with control group in a public university.

**METHODS:**

Using cephalometric analysis on lateral teleradiographs, the distance from the hyoid bone to the mandibular plane (MP-H) and the distance from the hyoid bone to the tuber (T-H) were correlated with the PAS measurement (airway) in two groups: 55 teleradiographs in the experimental group (with atypical deglutition) and 55 teleradiographs in the control group (normal deglutition). Both groups included subjects at the mixed dentition stage.

**RESULTS:**

The variable T-H presented a statistically significant correlation with PAS (0.0286) and the variable MP-H had a significant correlation with the variable PAS (0.0053). This positive correlation was significant only in the control group and not in the group with atypical swallowing.

**CONCLUSIONS:**

There was a positive correlation between the MP-H and PAS measurements and between the T-H and PAS measurements only in the group with normal swallowing. These correlations were not observed in the group with atypical swallowing.

## RESUMO

**CONTEXTO E OBJETIVO:**

Embora haja estreita relação entre respiração e deglutição, não existem estudos que avaliem a anatomia radiográfica de via aérea e sua possível correlação com a posição radiográfica do osso hioide. O objetivo deste estudo foi avaliar possível correlação da posição radiográfica do osso hioide e do espaço aéreo na radiografia lateral de crianças com deglutição atípica quando comparada com aquelas com deglutição normal.

**TIPO DE ESTUDO E LOCAL:**

Estudo transversal analítico com grupo controle em universidade pública.

**MÉTODOS:**

Por meio de análise cefalométrica em telerradiografias laterais, foi correlacionada a distância do osso hioide ao plano mandibular (MP-H) e do túber ao osso hioide (T-H) com a medida do espaço da via aérea (PAS) em dois grupos: 55 telerradiografias do grupo experimental (com deglutição atípica) e 55 telerradiografias do grupo controle (deglutição normal). Ambos os grupos incluíram indivíduos em fase de dentição mista.

**RESULTADOS:**

A variável T-H apresentou correlação estatisticamente significativa com PAS (0,0286) e a variável MP-H teve correlação significativa com a variável PAS (0,0053). Esta correlação positiva foi significativa apenas no grupo controle e não no grupo de deglutição atípica.

**CONCLUSÕES:**

Há correlação positiva entre as medidas MP-H e PAS e entre as medidas T-H e PAS somente no grupo de deglutição normal. Estas correlações não foram observadas no grupo de deglutição atípica.

## INTRODUCTION

Although swallowing is the first function to be established in the stomatognathic system, it is the last process to mature, because while the bone structures are growing and the dentition has not yet erupted, the tongue cannot acquire mature positioning and movement. Only when the child is around two years of age can an inconstant swallowing pattern that is transitional to the mature pattern (known as somatic swallowing) be expected, with the tongue at the limits of the dental arcade, the soft tissues more adjusted and the lips sealed. A visceral form of swallowing can persist well beyond the fourth year of life. However, it is then considered to be a dysfunction or abnormality because of its association with certain dental malocclusions and facial growing abnormalities.^1^^,^^2^ Such deglutition is classified as atypical.^3^^,^^4^^,^^5^

Recent studies have investigated the swallowing pattern in relation to child development and have concluded that atypical swallowing is present in half of the children examined at the age of three years, but changes significantly after the age of six years. Nevertheless, atypical swallowing is still present in 25 percent at the age of 12 years.^4^ The movements of the tongue during swallowing may be clinically assessed by asking the child to swallow liquids, semi-solids or solids, or even only saliva, to observe the protrusion of the tongue with the lips half-open or, if necessary, with lips opened with the fingers (forced opening method).^1^^,^^3^ By placing the hands on the masseters, it is possible to observe the presence or absence of contraction and to observe the ascendant movement of the hyoid bone under the thyroid cartilage. The participation of the perioral muscles is also observed, as well as whether the swallowing is loud, whether there is any retraction movement with the head, or whether any sign characterizing childlike swallowing is present.^1^^,^^3^^,^^4^^,^^5^ For a variety of reasons that so far remain incompletely explained, “infantile swallowing” may continue beyond the replacement of the deciduous teeth. Atypical deglutition has been attributed to sucking without nutritive purposes, use of feeding bottles, oral respiration, abnormalities of the central nervous system and anatomical abnormalities.^5^^,^^6^^,^^7^ However, there is no consensus regarding the etiology of atypical deglutition.^8^^,^^9^^,^^10^

Synchronization of sucking and swallowing is achieved through a close relationship between the muscles of the oral region, in order to generate suction pressure for opening and closing the mandible and for using the tongue for bolus formation and peristaltic transportation to the pharynx.^10^ During oral feeding, mechanical respiration involves appropriate activation of the diaphragm, intercostal muscles and muscles of the upper airways from the nose to the glottis.^10^ Among the likely anatomical abnormalities in cases of atypical deglutition is the positioning of the hyoid bone, since this is the origin or insertion point of several muscles relating to deglutition.^11^^,^^12^^,^^13^

Recent studies have evaluated the airway space and hyoid bone position in mouth breathing and obstructive sleep apnea (OSA).^14^^,^^15^^,^^16^^,^^17^ Although there is a close relationship between swallowing and breathing, there are no studies relating to the radiographic anatomy of the airway space in cases of atypical swallowing and its possible correlation with the radiographic position of the hyoid bone.

## OBJECTIVE

The objective of this study was to evaluate the possible correlation between the radiographic position of the hyoid bone and the airway space on lateral radiographs in children with atypical deglutition, in comparison with those with normal swallowing.

## METHODS

The research protocol for this study received unrestricted prior approval from the Research Ethics Committee of Faculdade de Medicina da Universidade Estadual de Campinas (FCM-Unicamp) (# 619/2005). This was a cross-sectional analytical study with a control group in which lateral teleradiographs from children of both genders at the phase of mixed dentition were evaluated. The whole study sample consisted of 110 teleradiographs in lateral view, from 52 female and 58 male subjects. The two groups were similar with regard to gender distribution. The mean ages of the control group (normal deglutition) and the experimental group were 9.46 years and 10.05 years, respectively. To define the control and experimental groups, an initial test using the forced opening method was conducted^1^^,^^2^^,^^3^ by three senior orthodontists simultaneously. The group to which the child’s teleradiograph should be allocated was defined by consensus.

Twenty lateral teleradiographs on 20 patients with a clinical diagnosis of atypical deglutition and another 20 lateral teleradiographs on 20 subjects with normal deglutition were selected for a pilot study in order to calculate the sample size. For this, the standard deviation of the control group and the difference between the means of the control and experimental groups were calculated. The alpha and beta values were 0.05 and 0.10 respectively. The size of the sample obtained through the calculation was 12 teleradiographs for measurements on the distance from the hyoid bone to the tuber (T-H), 35 for measurements on the distance from the hyoid bone to the mandibular plane (MP-H) and 26 for measurements on the airway space (PAS) in each group. A total of 55 teleradiographs were used, in order to also evaluate other variables within this research protocol that are not presented here.

At a significance level of 0.05, 110 teleradiographs (i.e. 55 in each group) were required to achieve a test power of 0.10. After sample size estimation, the whole sample was selected using the same criteria as in the pilot study, as described above.

**Figure 1.**
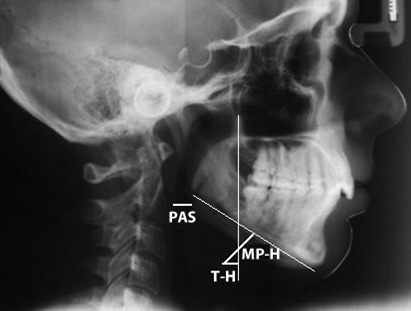
Cephalometric measurements: TH = tuber and MP-H = madibular plane.

All the lateral view teleradiographs selected for the present study were of dimensions 18 cm x 24 cm, and were obtained using the same Siemens apparatus for one second at 6 kVp, with a focal length of 1.5 meters. The examinations were performed with the patient’s head in a natural position (mirror position), and were performed by the same examiner. Using the selected lateral teleradiographs, cephalometric examination was performed in a darkened room with a negatoscope. An acetate sheet was laid over the teleradiograph and the following anatomoradiographic points and planes were marked on the sheet (Figure 1):

T-H: tuber (line of intersection between the center of the pterygomaxillary fissure and the posterior nasal spine) to hyoid (most anterosuperior point of the body of the hyoid bone);

MP-H: mandibular plane (line from the midpoint of the mandibular angle to the lowest point on the outline of the mentonian symphysis) to hyoid;

PAS: frontal wall of pharyngeal airway to posterior wall of pharyngeal airway.

Lateral teleradiographs that did not provide a good view of the anatomical structures used in the cephalometric examination were excluded from the study sample. Patients with dental agenesis, congenital poor orofacial formation or orthodontic and/or functional orthopedic treatment prior to the study, and those for whom there were doubts and imprecision regarding the diagnosis of deglutition, were also excluded. Lack of unanimity among the examiners regarding the clinical diagnosis was also a factor for exclusion from the sample. The patients’ skeletal pattern and any occurrences of malocclusion were not taken into consideration in this study.

The lateral teleradiographs from the experimental group and the control group were randomly put aside and numbered sequentially. The examiner performing the manual measurements was blinded to the patient data. The sequentially numbered teleradiographs were handed over to the examiner for the abovementioned standardized measurements to be made, and the results were recorded on a data-gathering instrument. To minimize systematic errors, the same examiner carried out data gathering on the whole sample on two occasions separated by a 20-day interval. After collection of radiographic data, age and sex data were added, along with whether atypical deglutition was present or not. On the other hand, all appropriate measures were taken to ensure confidentiality of the subjects’ personal data. Only the initials were recorded on the data-gathering instrument. There was no way in which anyone other than the investigator would be able to identify the individual to whom each teleradiograph belonged.

To investigate possible linear associations (correlations) between the MP-H, T-H and PAS variables, Spearman’s correlation analysis was performed. To investigate the intra-examiner consistency, the Wilcoxon test for related samples was used to detect possible differences between measurements obtained on two different occasions. The significance level used in the statistical tests was P = 0.05.

## RESULTS

The whole study sample consisted of 110 teleradiographs in lateral view, from 52 female and 58 male subjects. Only four teleradiographs were discarded because of the exclusion criteria. The two groups were similar regarding gender distribution (Table 1). The mean ages of the control group (normal swallowing) and the experimental group were 9.46 years and 10.05 years, respectively, without any significant difference between the groups (Table 2).

**Table 1.** Comparative analysis on the variable of male and female subjects

Deglutition	Male	Female	P-value from chi-square test
Normal	33	22	0.1266
Atypical	25	30	

**Table 2.** Comparative analysis of the age variable (years)

Deglutition	n	Mean	Standard deviation	Minimum	Median	Maximum	P-value from Mann-Whitney test
Normal	55	9.32	1.83	6.41	9.25	11.66	0.6345
Atypical	55	9.58	2.13	6.41	9.08	11.91	

To investigate the intra-examiner consistency, the Wilcoxon test for related samples was used to detect possible differences between measurements obtained on two different occasions. However, no significant difference was found between these two measurements. The Mann-Whitney U test was used to compare the two groups regarding the cephalometric measurements. The average distance of the MP-H variable was 11.69 millimeters for the control group and 16.14 millimeters for the experimental group, with a statistically significant difference (P = 0.016) (Table 3).^8^ The average distance of the T-H variable was 2.26 millimeters for the control group and -5.89 millimeters for the experimental group, with a significant difference (P < 0.001) (Table 4).^8^

**Table 3.** Comparative analysis on the MP-H variable (mm)

Deglutition	n	Mean	Standard deviation	Minimum	Median	Maximum	P-value from Mann-Whitney test
Normal	55	11.69	5.13	3.00	12.00	21.00	0.016
Atypical	55	16.14	4.86	7.00	16.00	27.00	

**Table 4.** Comparative analysis on the T-H variable (mm)

Deglutition	n	Mean	Standard deviation	Minimum	Median	Maximum	P-value from Mann-Whitney test
Normal	55	2.26	1.79	0.00	2.00	6.00	< 0.001
Atypical	55	-5.89	4.77	-16.00	-5.00	4.00	

The average distance of the PAS variable was 7 mm in the experimental group and 10 mm in the control group, with a statistically significant difference (P < 0.001) (Table 5).^18^ There were positive correlations between MP-H and PAS (P = 0.0053) and T-H and PAS (P = 0.0286), but these correlations were only observed in the control group (Table 6).

**Table 5.** Comparative analysis on the PAS (airway) variable (mm)

Deglutition	n	Mean	Standard deviation	Minimum	Median	Maximum	P-value from Mann-Whitney test
Normal	55	10.53	2.43	5.00	10.00	15.00	< 0.001
Atypical	55	7.82	2.93	3.00	7.00	13.00	

**Table 6.** Spearman’s linear correlation coefficients between the variables in each group

	Normal deglutition	Normal deglutition	Atypical deglutition	Atypical deglutition
PAS	T-H	MP-H	T-H	MP-H
	0.29536	0.37103	-0.19513	0.01967
	0.0286^*^	0.0053^*^	0.1534	0.8867

^*^Significant correlation of moderate intensity: P-value < 0.05.

## DISCUSSION

The study presented here shows that there was a significant difference in the radiographic size of the PAS measurement between the study groups, such that it was smaller in the group with atypical swallowing. There were also significant differences in the T-H and MP-H measurements, such that in the group with normal swallowing, the hyoid bone was radiographically closer to the mandibular plane and to the T line (tuber). In assessing the possibility of a correlation between the PAS and MP-H variables and between the PAS and T-H variables, it could be seen that there was a positive correlation in radiographic position between the hyoid bone and the PAS measurement only in the group with normal swallowing.

The PAS measurement has been described as the distance between the posterior and anterior parts of the pharynx, where the base of the tongue is located.^12^^,^^18^ This leads us to believe that the shortening of the airways in patients with atypical swallowing might cause changes in tongue positioning, which would lead to changes in the position of the hyoid bone.

Craniofacial abnormalities in children with respiratory obstruction have been studied over recent years. However, the absence of a direct relationship between the cause of respiratory obstruction and its effect on craniofacial growth has led to considerable controversy in the literature.^11^^,^^12^^,^^13^^,^^14^^,^^15^ The most widely accepted theory is that tonsil hypertrophy, which leads to pharyngeal obstruction, causes mouth breathing^11^ and changes in the child’s way of positioning the orofacial muscles and mandible. These changes, in turn, influence mastication, swallowing and phonation, and lead to occlusal and skeletal abnormalities.^11^^,^^16^

The MP-H variable has been used in cephalometric studies in relation to obstructive sleep apnea and hypopnea syndrome (OSAHS).^13^^-^^14^ The data from these studies are similar to the results from our study, in that they show that in OSAHS, the hyoid bone is more distant from the mandibular plane, which was also observed in our study on atypical swallowing. This observation leads us to believe that the hyoid bone is perhaps related to maintaining and stabilizing the airway. We also believe that the lower position of the hyoid bone in the group with atypical swallowing may have been caused by a change in the suprahyoid and infrahyoid muscles, and possibly hypertonia of the infrahyoid and hypotonia of the suprahyoid muscles. These changes to traction may have been responsible for the abnormal position of the tongue in cases of atypical swallowing,^1^ which is a lower position than in cases of normal swallowing.

The T-H variable was originally used in this study with the intention of observing the anterior-posterior positioning of the hyoid bone in relation to the face. We took the descendant line of the pterygomaxillary fissure to the level of the hyoid bone as mark zero. Distances to its right were measured as positive values and distances to its left were taken to be negative values. Therefore, the negative value of the T-H variable found in the group with atypical swallowing refers to the more posterior position of the hyoid bone, in relation to the descendant line of the pterygomaxillary fissure. One hypothesis that has already been studied is that the radiographic position of the hyoid bone is dependent on facial type and is associated with factors such as age, obesity, breathing and apnea,^1^^,^^3^^,^^17^ Our results demonstrated that functional alterations such as atypical swallowing may also be among the adjunct factors that alter the position of the hyoid bone.

Recent studies^7^^,^^10^ have correlated the pharynx measurement with mandibular position and have suggested that with mandibular advancement there is an increase in PAS measure. New knowledge of the correlation between the measurements studied in the normal swallowing group may corroborate this hypothesis. Because there are muscles connecting the hyoid bone to the mandible and because the hyoid is a mobile bone, the position of the hyoid may be altered because of the position of the mandible. This may be the reason why we only found a correlation between the measurements studied in the normal swallowing group. In the atypical swallowing group, the hyoid bone was in a more inferior and posterior position, which would indicate mandible positioning that was more posterior, with consequently decreased airway size. We believe that the decrease in the PAS measurement may be related to a more posterior position for the mandible, caused primarily by the lower positioning of the tongue, thereby causing a decrease in the PAS measurement. In the group with atypical swallowing, we did not observe this correlation, since the hyoid bone is more distant from the mandibular plane and from the pterygomaxillary line.

Clinical tests used for defining atypical swallowing have limitations, and the final diagnosis of the type of swallowing is based on each examiner’s experience, through assessing the involvement of the orbicular muscles and other compensatory components, in swallowing. Perfect sealing of the oral cavity and contraction of the masseter muscle, which helps the dental occlusion and is necessary for swallowing, was observed in individuals with normal swallowing. Lip incompetence and perioral muscle exertion to help in swallowing, and, in some cases, spilling of content from the labial commissure and interposition of the tongue to help in relation to incompetent lip sealing have been observed in cases of atypical swallowing.^1^^,^^3^^,^^5^^,^^14^

This radiographic study evaluated the relationships between known parameters, but these relationships were used in a novel manner, in that they were correlated with normal and abnormal deglutition. In addition to studying a normal group, this study also evaluated patients with atypical deglutition, which is a moderately prevalent clinical condition that can have an impact on orofacial, nutritional, esthetic and psychosocial development.^13^ Since deglutition is a highly complex and coordinated function, it requires activation of many anatomical structures relating to the tongue. Insufficient functional stimulation of the stomatognathic system, especially the tongue, might be the main factor in persistence of childlike deglutition.^1^ Therefore, because pediatricians are the first professionals to have contact with such children, they should always be aware of the degree of maturation of swallowing function and refer such children to a dentist when maxillary-mandibular changes are noticed.

## CONCLUSION

There are positive correlations between the MP-H and PAS measurements and between the T-H and PAS measurements only in the group with normal swallowing. These correlations were not observed in the group with atypical swallowing.
